# Management of Apical Third Root Fractures in Radiculomegaly of Mandibular Anterior Teeth: A Rare Case Report

**DOI:** 10.7759/cureus.55012

**Published:** 2024-02-27

**Authors:** Navdeep Jethi, Karan Maheshwari, Khushdeep Kaur

**Affiliations:** 1 Conservative Dentistry and Endodontics, Daswani Dental College and Research Centre, Kota, IND; 2 Orthodontics and Dentofacial Orthopaedics, Adesh Institute of Dental Sciences and Research, Bathinda, IND; 3 Dentistry, Rayat Bahara Dental College and Hospital, Mohali, IND

**Keywords:** apical third, dental root fracture, primarycare, mandibular anterior teeth, root canal working length, dental anatomy, dental trauma

## Abstract

In this case report, two uncommon fractures of the root located at the apex of the mandibular anterior teeth region were successfully treated and preserved using non-surgical endodontic procedures. These teeth possess a unique size, characterized by the presence of exceptionally elongated lateral incisors measuring approximately 29 mm, central incisors measuring approximately 25 mm, and canines measuring approximately 30 mm. For the lower left central incisor, the root canal was carefully navigated to the coronal fragment, whereas the apical fragment was left undisturbed and preserved. In the case of the remarkably elongated lower right lateral incisor, the process of 'unification' between the apical and coronal fragments was accomplished through the utilization of Gutta-percha and Ah plus sealer. Subsequently, the mandibular anterior teeth were stabilized and immobilized using dental splints for a duration of one and a half months, with regular follow-ups conducted over a span of six months and one year, during which positive healing outcomes were observed.

## Introduction

Anterior teeth are highly prone to fractures in cases of trauma [1]. The prevalence of horizontal root fractures in dental trauma is very low (1.2%-7%), and horizontal root fractures of the apical third are rare [1]. The chances of apical root fracture of the mandibular lateral incisors are very rare among other anterior teeth [2]. As the average overall length of these teeth is approximately 22 mm, an overall length of 29 mm is rare for mandibular lateral incisors [3]. Therefore, the present case is rare with respect to the unusual length of the mandibular lateral incisors and the apical third root fracture in the horizontal section.

The root length of a tooth and the level of the fracture line play important roles in the treatment plan and prognosis of a root fracture episode [4]. Variations in root length and root canal morphology are often observed in endodontics [4]. As per Rotstein et al., the average overall length of the mandibular central incisors and mandibular lateral incisors ranges are 19.4-23.6 mm and 20.2-24.6 mm, respectively, with a 60% possibility of straight canals in mandibular lateral incisors, and the labial curvatures are also observed [4]. The prevalence of distal curvature in these teeth is as high as 23%. In non-extraction scenarios, the overall length of the tooth can be estimated or recorded using intraoral and digital radiography [4,5]. In addition, working length estimation aids in endodontics can be used to record the overlength of a tooth [5].

Apical third horizontal tooth fractures are generally not diagnosed in the absence of radiography. These fractures pose very little threat to the prognosis of teeth and sometimes require no treatment [1]. Endodontic treatment of coronal fragments is indicated in cases of pain or mobility, leaving the apical fragment intact. Surgical removal of the apical fragment is rarely performed in patients with severe and persistent pain [4].

The current case report was exceptional because of the atypical dimensions of the mandibular lateral incisors (< 28.30 mm) and horizontal root fracture in the apical third of the right mandibular lateral incisor and left mandibular central incisor. Pain management was executed using an innovative noninvasive approach, which was subsequently accompanied by immobilization of the anterior teeth to address the issue of mobility.

## Case presentation

Patient history

**Figure 1:**
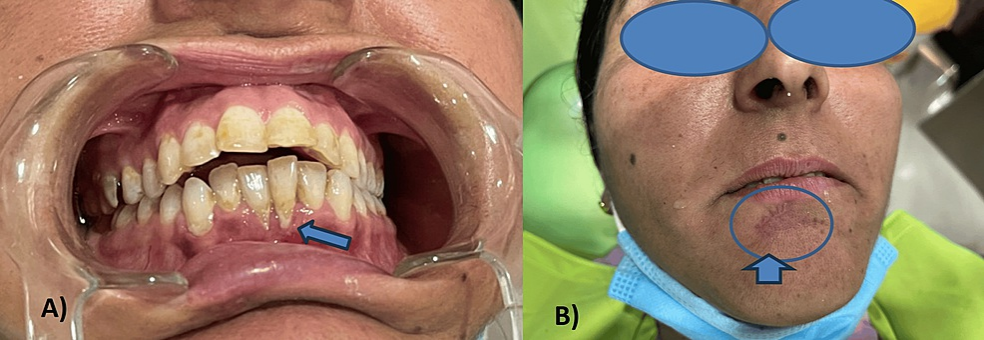
Preoperative photographs A) Gingival recession in 31 and 41 and B) extraoral suture scar (blue circle)

A calf collided with the chin of a 42-year-old female who presented before a medical facility with a complaint of swelling over the face and difficulty opening the mouth. The direct impact on the mandibular anterior dentition and lacerations in the lower lip regions of the patient resulted from a direct blow to the lower jaw, chin, and lower lip regions. At that time, the patient was not fully able to open the mouth due to soft tissue swellings and lacerations over the lower lip. She was treated at a primary health care center before she saw her dentist. Measures to halt bleeding from the affected area, initial medical assistance, and suturing were performed at a primary healthcare center in the rural community a few days prior. Inflammation remained prominent for a week. The patient was prescribed antibacterial and anti-inflammatory medications and was scheduled for a follow-up appointment after suture removal. After a week, the patient returned with a relapse of facial swelling, normal mouth opening, and jaw movements. Wounds below the lower lip and chin area are healed now. Scars could be seen under the lower lip, but there was pain in the mandibular anterior teeth, for which she was referred for a dental diagnosis (Figure 1).

Clinical examination

The patient's medical and dental history was evaluated, with particular attention given to COVID-19 history [6]. The patient exhibited a body temperature of 36.6 °C and a normal oxygen saturation level of < 96%, while no other symptoms were observed. Subsequently, oral and written consents were obtained from the patient. The protocols pertaining to COVID-19 were adhered to, with endodontists and attendants employing appropriate barrier techniques, such as face shield protection, double-masking, and gloving. Prior to any oral intervention, the patient was instructed to rinse with chlorhexidine mouthwash. The chief complaint was pain and mobility of the anterior mandibular teeth. It was not a mandibular fracture, and at this time, the patient had no disability in mouth opening or jaw movements. She was able to open the mouth by then; it was three finger lengths. The central and lateral incisors demonstrated tenderness upon percussion, and grade one mobility was observed in the lower left central and right lateral incisors. Additionally, periodontal pockets accompanied by generalized plaque and calculus were present, along with recession in both the lower central incisors. Dental pulp exhibited a positive vitality test, as confirmed by pulp testing. The patient reported sharp pain that had interrupted their sleep during the night, which was alleviated by medication but persisted every six to eight hours.

Radiographic findings

**Figure 2:**
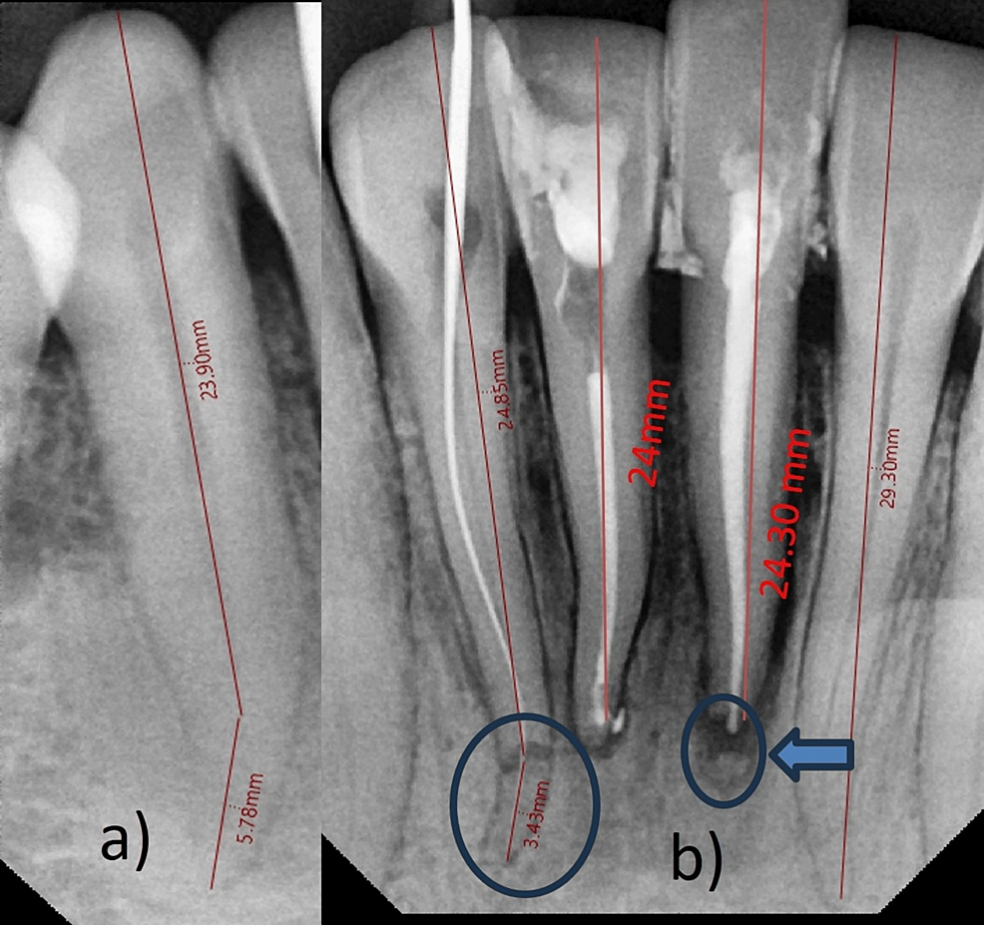
Radiographic interpretations of rarely large mandibular anterior teeth a) Right lower canine length = 30 mm. b) Left lower lateral incisor of 29 mm and lower right lateral incisor of 28.3 mm, and horizontal apical third fracture in the lower right lateral incisor (blue circle); right lower central incisor of 24 mm and left lower central incisor of 25 mm (coronal segment = 24.3 and apical segment of 0.7 mm), with apical third fracture (blue arrow and circle)

Radiographs were taken with a Vatech intraoral sensor and viewed using EZ Dent sensor software (Vatech India Pvt. Ltd., New Delhi, India) (Figure 2).

Radiographic analyses revealed a horizontal fracture in the apical third of the root of the lower right lateral incisor (tooth 42), which exhibited a distal curvature at the apical third. Additionally, the root length was greater than the typical length, with the apical fragment measuring 3.43 mm and the coronal fragment measuring 24.85 mm. The lower right central incisor (tooth 41) was found to have a periapical injury (< 0.5 mm) at the apex of the root with an overall length of 24 mm. Furthermore, a small horizontal fracture in the apical third of the root was identified in the lower left central incisor (tooth number 31), accompanied by a widening of the periodontal ligament. The apical fragment of this tooth measured 0.7 mm, while the coronal fragment measured 24.3 mm, resulting in an overall length of 25 mm. In contrast, the lower left lateral incisor (tooth 32) had a longer overall length (29.3 mm) and distal dilation. Finally, the mandibular canines on both sides (teeth 43 and 33) measured approximately 30 mm.

According to the Vertucci classification, the canal anatomy in 42 and 43 was type iii, with one canal leaving the pulp chamber, dividing into two within the middle, and merging to exit as one canal [3].

Treatment plan

There was negligible luxation in the mandibular anterior teeth 31 and 42. The radiographs reveal the apical third fractures of teeth 42 and 31. For the treatment plan in 31, as the size of the apical fragment was too small, about 0.7 mm, technically very close to the apex, we generally keep the obturation 0.5-1 mm short of the periapex, so the treatment of the coronal segment was enough to relieve pain. However, in 42, the size of the apical fragment was 3.43 mm, and in the case of pulp remnants troubling the patient with pain, it was necessary to clean the apex to avoid traditional surgical removal of the apical fragment, followed by splinting for a month. In horizontal root fractures, mobility and dislocation of tooth fragments can cause pulp necrosis and periodontal pathologies, so stabilizing the tooth is necessary for healing. Additionally, approximation and unification of the root fragments were planned for 42 for the same reason later on.

The treatment plan has been mentioned in Table 1.

**Table 1:** Treatment plan

COVID-19 prevention	Thorough COVID-19 history-taking, mouthwash rinses for patients before treatment initiation, proper barrier technique for endodontists and attendants
Periodontal treatment	Scaling (before endodontic treatment) and splinting (after endodontic treatment)
Endodontic treatment	Root canal therapy for 41, apical third fracture management for 31, apical third root fracture management for 42

**Figure 3:**
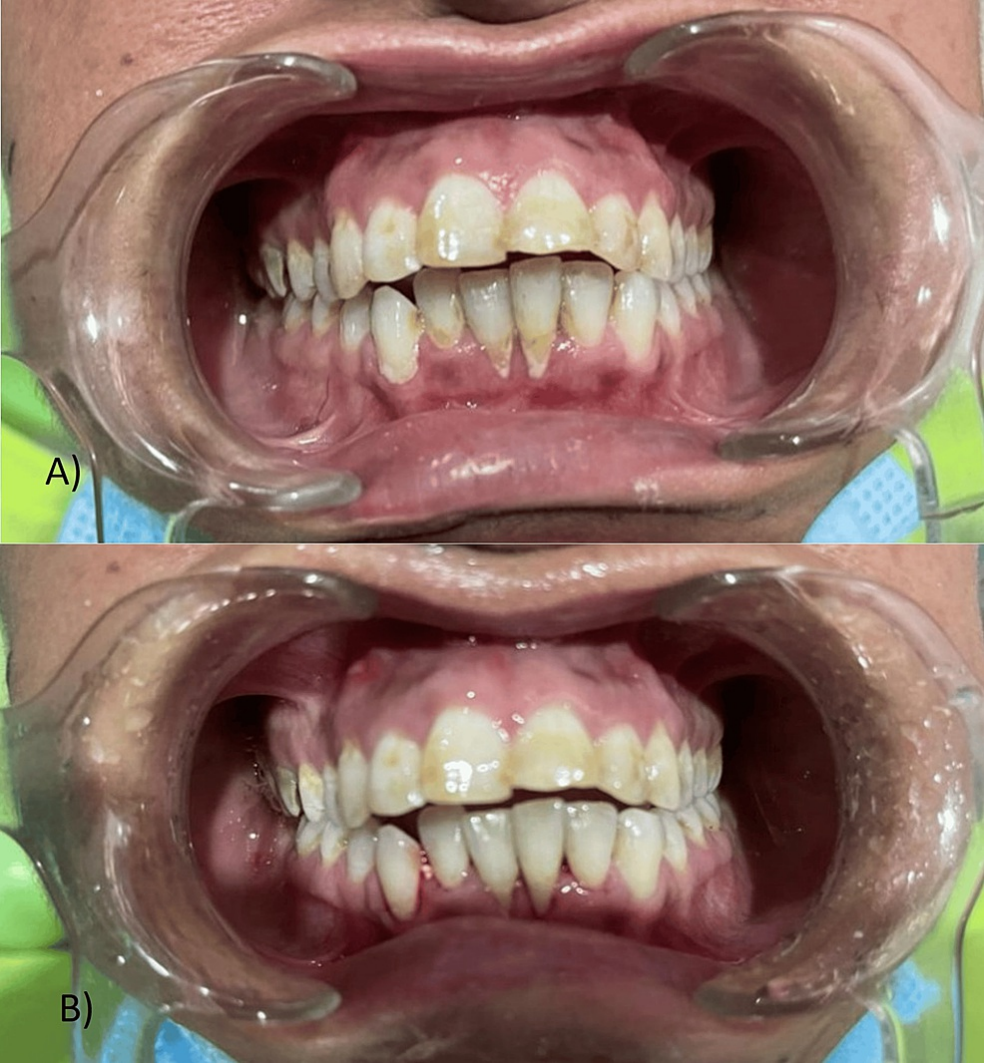
Ultrasonic scaling for mandibular anterior teeth: A) before scaling and B) after scaling

Periodontal intervention was administered before the initiation of endodontic intervention, encompassing thorough oral prophylaxis, as the presence of bacterial microflora had the potential to impede the efficacy of the endodontic procedure. Calculi and recession were observed in the vicinity of teeth 31 and 41, concomitant with the manifestation of fluorosis (Figure 3). Ultrasonic scaling was done to clean the plaque and calculus in the mandibular anterior teeth, which may interfere with the healing of small pockets around them.

**Figure 4:**
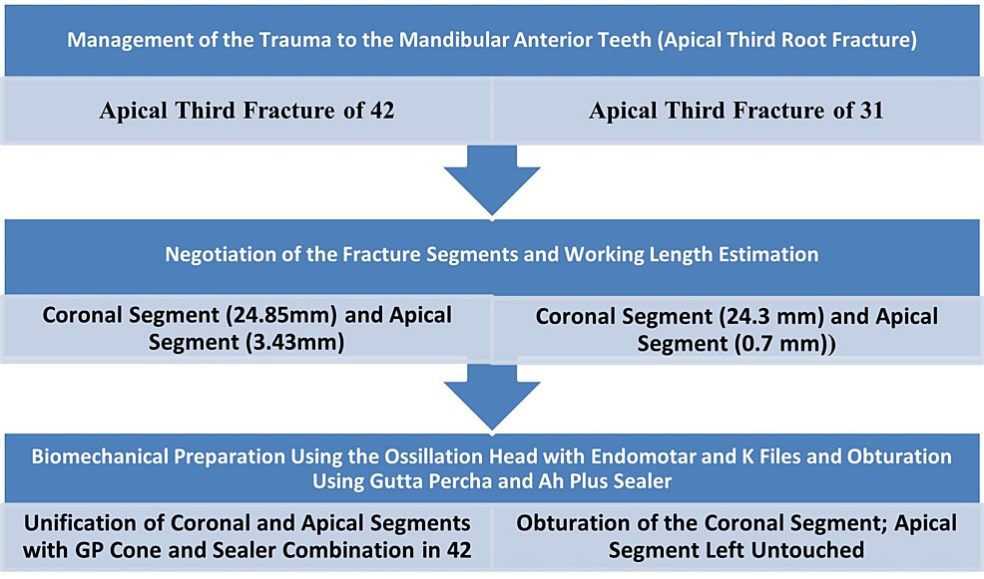
Management of trauma to the mandibular anterior teeth (apical third root fracture)

Endodontic treatment was performed for apical third root fractures in tooth 31 and tooth 42, respectively. Two different approaches for the non-surgical management of trauma in both teeth were applied (Figure 4).

**Figure 5:**
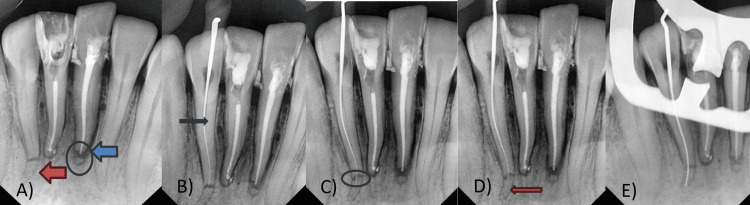
A) Root canal treatment in 31 and 41, apical fractured fragment left untouched in 31 ( circle and blue arrow) and apical third root fracture in 42 (red arrow). B) Coronal segment negotiation in 42 (black arrow). C) Fracture line in 42 (circle). D) Apical segment negotiation in 42 (red arrow). E) Coronal to apical segment access in 42

The methods were as follows, in the subsequent manner: in the primary technique, the apical fragment was left untouched in 31. Initially, endodontic treatment was scheduled for 31 and 41, as greater pain was present in these teeth. The patient was anesthetized with lignocaine (1:80000), and rubber dam isolation was performed. Access opening was performed for both central incisors, and a triangular access cavity was prepared using round and Endo Z burs. The pulp was removed; initial negotiations were conducted with 6 K, 8 K, 10 K, and 15 K files; and a glide path was established. The working length was estimated using digital radiographs and an apex locator (31 = 25 mm and 41 = 24 mm). The canals were enlarged up to 30 K using lubricants (ethylenediaminetetraacetic acid gel) with combined irrigation with 2.5% sodium hypochlorite (NaOCl) and normal saline. Biomechanical preparation was performed using K files. An oscillating head with an endomotor handpiece was used. A calcium hydroxide dressing was applied during the initial appointment. In the subsequent appointment, obturation was performed using GuttaPercha and AH Plus Sealer (Dentsply). Hot vertical compaction was performed by using a fast-pack obturation pen. The lower right central incisor exhibited a lateral opening and labial curvature, and a sealer puff was observed at the same apex. The root canal filling in the lower left central incisor was approximated up to a length of 24.3 mm until the coronal segment of the tooth and apical fragment were not touched. Post-endodontic restoration was accomplished using glass ionomer cement (GIC) (Figures 5A-5B).

**Figure 6:**
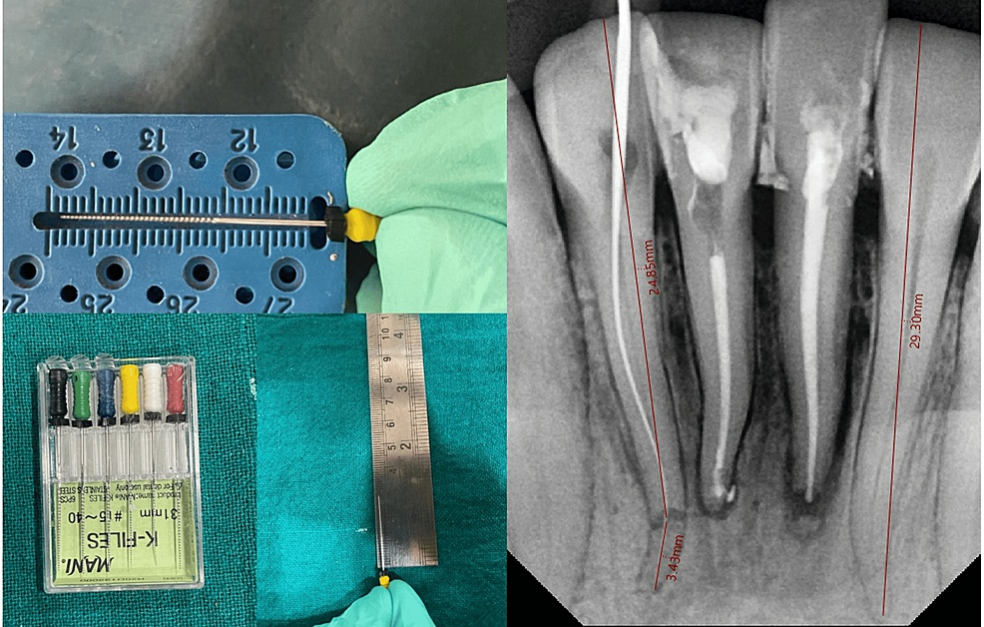
Working length estimation: 32 = 28.5 mm and 42 = 29.3 mm A) Using endo-gauge, 31 mm K files. B) Using VATECH RVG software

The second approach utilized a non-surgical method to approximate the coronal and apical fragments in tooth number 42. Initially, the plan for tooth number 42 involved an observational strategy. However, due to the patient's reported discomfort, pain, and increased tooth mobility in tooth number 42, it was decided to proceed with a non-surgical endodontic procedure to reattach the coronal fragment of the tooth. The necessary precautions for COVID-19, patient consent, and barrier techniques were followed accordingly. The patient was administered anesthesia, and rubber dam isolation was performed. An access cavity was created, and the pulp was removed from the coronal fragment. K files measuring 25 mm in length were used to establish an initial negotiation and glide path in the coronal segment. The negotiation and preparation of the coronal segment involved the use of a combination of K files and an oscillating head attached to the endomotor handpiece (NSK). Initially, only the coronal fragment was prepared, with straight-line access obtained, and a calcium hydroxide dressing was applied. The curved apical segment was left untouched. After duration of three days, the patient experienced a recurrence of endodontic pain, which did not respond to medication. Subsequently, the radiographs were examined again. The presence of residual pulp in the apical third fragments might explain the persistence of pain. The utilization of 31 mm files facilitated the negotiation of the apical fragments, while a straight-line approach was employed to access the coronal to apical fragment of the root. The balance force technique was employed in a crown-down manner to ensure proper shaping. The working length was determined using apex locators and radiographic interpretations and was measured on an endo-gauze scale. The working length of this particular tooth was approximately 28.30 mm, as shown in Figure 6.

**Figure 7:**
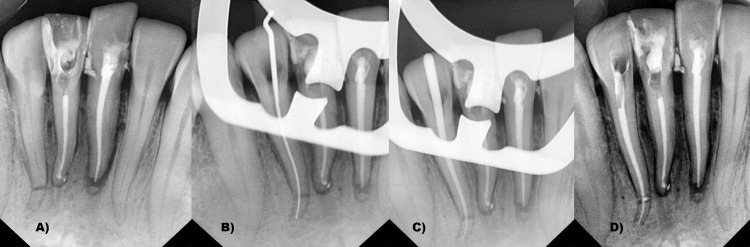
Endodontic therapy and unifying apical and coronal segments in apically fractured 42 A) Preoperative radiograph; B) working length estimation; C) master cone; D) unifying apical and coronal segments

The process of enlarging the canals involves utilizing a series of K files, including 10 K, 15 K, 20 K, 25 K, and 35 K. To perform this procedure, an oscillating head was employed in conjunction with the Endomate DT endomotor operating at a speed of 40 and a torque of 0 for all files. After utilization of each file, ethylenediaminetetraacetic acid (EDTA) lubrication, 2.5% sodium hypochlorite, and normal saline irrigation were administered. Gutta-percha and AH Plus sealers were used for the obturation process. The warm vertical compaction was performed using an obstruction pen. Finally, the post-endodontic restoration was completed using the GIC, as depicted in Figure 7.

**Figure 8:**
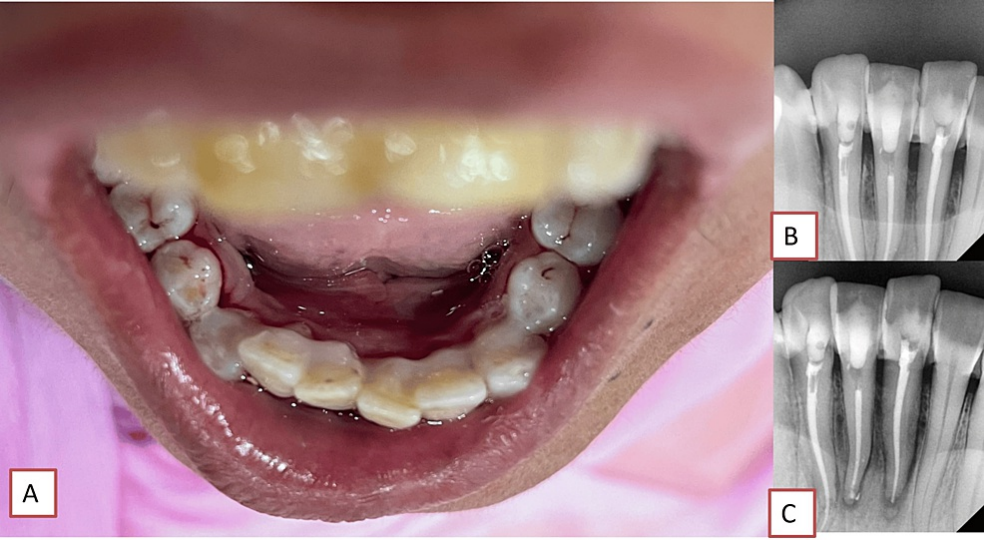
Composite splinting in mandibular anterior teeth A) Postoperative intraoral photograph. B) and C) Splinting radiographs

**Figure 9:**
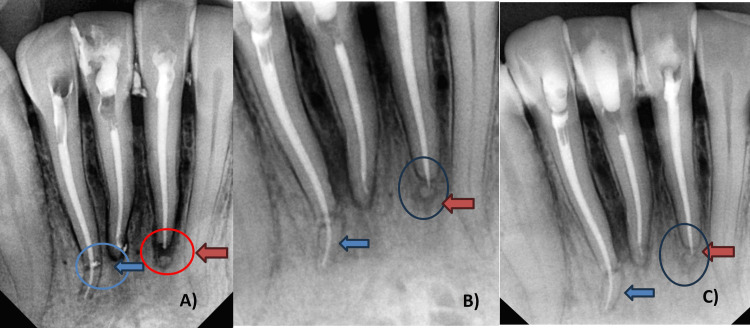
Follow-up radiographs A) Immediate post-obturation radiograph (blue circle and arrow show unified segments in 42, and red circle and arrow show obturation in 31). B) After six months (blue arrow in 42 and red arrow with circle in 32 show positive healing). C) After one year (red arrow and circle in 31 and blue arrow in 42 show positive healing)

A composite splint was used to stabilize the teeth in the dental arch. The flowable composite was used to splint the teeth together, specifically targeting the mesial side of the first premolars from the left side to the right side. The objective of this splinting procedure is to immobilize teeth and facilitate healing. The patient was scheduled for follow-up appointments after 1.5, six months, and one year (Figures 8-9).

## Discussion

**Figure 10:**
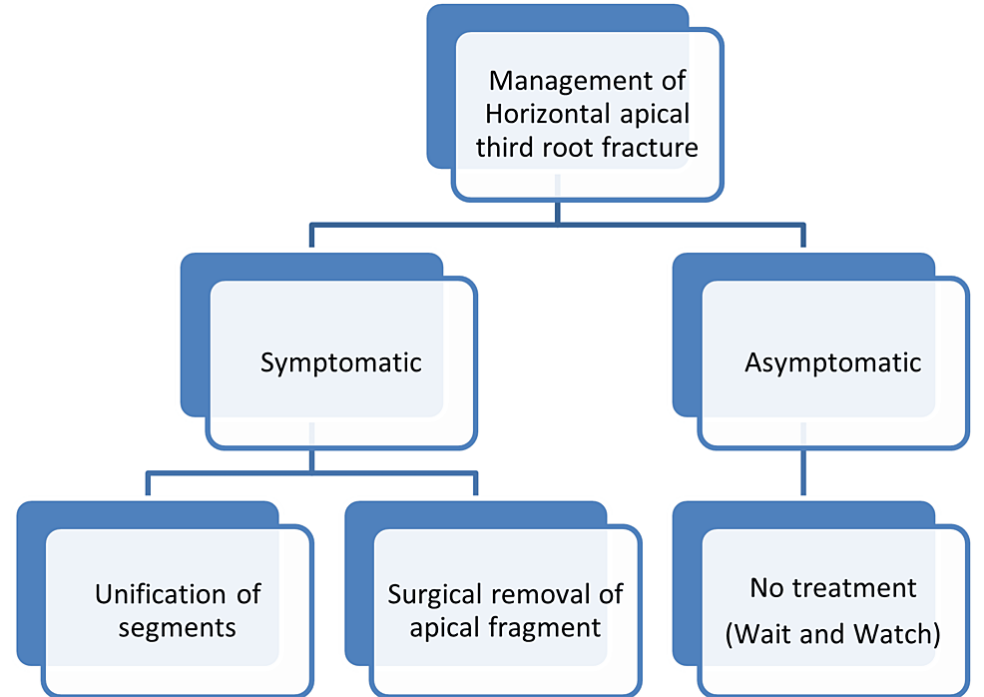
Jethi’s classification for the management of apical third root fracture (based on symptomatic conditions)

Direct tooth trauma, which is concentrated over a small area of the tooth, is the primary cause of horizontal tooth root fractures [1]. The probability of horizontal fracture of tooth roots is generally very low, ranging from 1.2% to 7% [2]. The lower anterior teeth are rarely involved in traumatic injuries of the mouth; of these, mandibular lateral incisors are the least recorded for root fractures, and apical third root fractures are rare again [2]. In this case, long, distally curved mandibular lateral incisors with an apical third fracture were rarely recorded, along with an unusual canine length of 30 mm. In middle-third fractures, approximation of the coronal and apical fragments is generally performed using the post and core [2]. Mostly, no treatment is indicated for apical third root fractures; a wait-and-watch strategy is indicated [3]. Management of apical third root fractures depends on the symptomatic or asymptomatic status of the tooth [3-4]. In symptomatic cases, non-surgical treatment includes endodontic treatment of the coronal fragment only; surgical removal of the apical fragment is indicated if persistent pulp necrosis is the cause of the patient’s arrival [3-4] (Figure 10). In an apical third root fracture, the displacement of the coronal fragment may cause difficulty in the approximation of the two fracture fragments. Sometimes enough luxated fragments limit the chances of coronal and apical fragment approximation, hence non-surgical unification. The teeth, in this case, were slightly subluxed, which reduced the limitation for the unification of fragments [4].

Horizontal root fracture management depends on the level of the root fracture [7]. The root of each tooth was divided into three levels: the coronal, middle, and apical thirds. Coronal third fractures generally indicate poor prognosis and extraction [7,8].

Apex locators and RVG can accurately record the working length, that is, the overall length of a tooth [5]. In this case, non-surgical endodontic pain management for apical third root fracture in an approximately 29-mm-long mandibular lateral incisor was chosen. The tooth was symptomatic, and the pulp in the apical fragment had troubled the patient. Unification of the coronal and apical fragments of the tooth root was performed using gutta-percha and a resin-based sealer. In addition, traditional non-surgical endodontic treatment with approximately 25-mm-long mandibular central incisors was performed. The coronal fragment was negotiated and endodontically treated because the apical fragment was small, and it did not create any post-obturation pain. The patient remained comfortable and asymptomatic for six months, and no persistence of infection was observed on radiographs. The patient was instructed to attend follow-ups after one year. Additionally, endodontic radiography is the best method to use as a forensic record, as the variations and unique features of dentition, such as root fractures, can be preserved through the use of digital radiography [9,10].

## Conclusions

Generally, apical fragments remain undisturbed during episodes of apical third root fractures when they exhibit no symptoms. In situations where teeth exhibit atypical root lengths, the decision to perform non-surgical endodontic unification of the coronal and apical fragments in root apical third fractures may be warranted depending on the size of the apical portion and the presence of symptomatic pulp remnants. This approach facilitates the alleviation of endodontic pain and reduces the likelihood of surgical intervention to remove the fractured apical fragments.

## References

[1] Abbott PV (2019). Diagnosis and management of transverse root fractures. J Endod.

[2] Karhade I, Gulve MN (2016). Management of horizontal root fracture in the middle third via intraradicular splinting using a fiber post. Case Rep Dent.

[3] Garg N, Garg A (2014). Textbook of Endodontics.

[4] Rotstein I, Ingle JI (2019). Ingle’s Endodontics. 7th Edition. Int J Clin Pediatr Dent.

[5] Krishna VP (2020). Grossman's Endodontic Practice. 14th Edition. Int J Clin Pediatr Dent.

[6] Jethi N, Pandav G, Nagri D, Pandav S, Kumari D, Kaur M (2020). Asymptomatic COVID-19 patients and possible screening before an emergency aerosol related endodontic protocols in dental clinic-a review. J Family Med Prim Care.

[7] Welbury RR, Kinirons MJ, Day P (2002). Outcomes for root-fractured permanent incisors: a retrospective study. Pediatric Dentistry.

[8] Santos KS, Miranzi MAS, Miranzi BAS (2016). Horizontal root fracture in the apical third: case report. Rev Gaúch Odontol.

[9] Shanmugaraj M, Nivedha R, Mathan R, Balagopal S (2007). Evaluation of working length determination methods: an in vivo / ex vivo study. Indian J Dent Res.

[10] Jethi N, Arora KS (2020). Forensic endodontics and national identity programs in India. Indian J Dent Res.

